# Extraction, Characterization and Incorporation of *Hypericum scruglii* Extract in Ad Hoc Formulated Phospholipid Vesicles Designed for the Treatment of Skin Diseases Connected with Oxidative Stress

**DOI:** 10.3390/pharmaceutics12111010

**Published:** 2020-10-23

**Authors:** Mohamad Allaw, Maria Manconi, Matteo Aroffu, Francesca Marongiu, Marco Porceddu, Gianluigi Bacchetta, Iris Usach, Rita Abi Rached, Hiba N. Rajha, Richard G. Maroun, Jose Luis Pedraz, Tania B. Lopez-Mendez, Anna Maria Fadda, Maria Letizia Manca

**Affiliations:** 1 Department of Life and Environmental Sciences, Drug Science Division, University of Cagliari, 09124 Cagliari, Italy; allaw.mohamad.22@gmail.com (M.A.); matteo.aroffu@gmail.com (M.A.); fmarongiu@unica.it (F.M.); amfadda@unica.it (A.M.F.); mlmanca@unica.it (M.L.M.); 2 Sardinian Germplasm Bank (BG-SAR), Hortus Botanicus Karalitanus (HBK), University of Cagliari, Viale S. Ignazio da Laconi, 9-11, 09123 Cagliari, Italy; porceddu.marco@unica.it (M.P.); bacchet@unica.it (G.B.); 3 Centre for the Conservation of Biodiversity (CCB), Department of Life and Environmental Sciences, University of Cagliari, Viale S. Ignazio da Laconi 11-13, 09123 Cagliari, Italy; 4 Department of Pharmacy and Pharmaceutical Technology and Parasitology, University of Valencia, Burjassot, 46100 Valencia, Spain; Iris.Usach@uv.es; 5 Centre d’Analyses et de Recherche, Unité de Recherche TVA, Laboratoire CTA, Faculté des Sciences, Université Saint-Joseph, B.P. 17-5208 Riad El Solh, Beirut 1104 2020, Lebanon; rita.abirached@net.usj.edu.lb (R.A.R.); hiba.rajha@usj.edu.lb (H.N.R.); richard.maroun@usj.edu.lb (R.G.M.); 6 NanoBioCel Group, University of Basque Country, Paseo de la Universidad 7, 01006 Vitoria, Spain; joseluis.pedraz@ehu.eus (J.L.P.); tblopez01@gmail.com (T.B.L.-M.); 7 Networking Research Centre of Bioengineering, Biomaterials and Nanomedicine (CIBER–BBN), Av. Monforte de Lemos, 3-5. Pabellón, 28029 Madrid, Spain; 8 Ecole Supérieure d’Ingénieurs de Beyrouth (ESIB), Saint-Joseph University, CST Mkalles Mar Roukos, Riad El Solh, Beirut 1107 2050, Lebanon

**Keywords:** Hypericaceae, phospholipid vesicles, glycerosomes, gelatin, hyaluronan, oxidative stress, scratch assay, keratinocyte uptake

## Abstract

An extract of *Hypericum scruglii,* an endangered endemic plant of Sardinia (Italy), was prepared and characterized. It was loaded in special phospholipid vesicles, glycerosomes, which were modified by adding maltodextrin (glucidex) and a polymer (gelatin or hyaluronan). The corresponding liposomes were also prepared and used as reference. The vesicles disclosed suitable physicochemical features for skin delivery. Indeed, their mean diameter ranged from 120 to 160 nm, they were homogeneously dispersed (polydispersity index ≤ 0.30), and their zeta potential was highly negative (~−45 mV). The vesicle dispersions maintained unchanged characteristics during 60 days of storage, were highly biocompatible, and were able to protect keratinocytes against damages due to oxidative stress induced by treating them with hydrogen peroxide. Vesicles were also capable of promoting cell proliferation and migration in vitro by means of a scratch wound assay. The results confirmed the fruitful delivery of the extract of *H. scruglii* in glycerosomes modified with glucidex and gelatin and their promising ability for skin protection and treatment.

## 1. Introduction

The skin represents the main barrier of our body and it is specifically designed to perform different functions such as protection from various external insults, maintenance of the internal homeostasis, and sensory perception [1]. Indeed, it protects the body from various daily challenges, avoiding excessive loss of water and providing a defense against mechanical insults, passage of xenobiotics, and absorption of chemicals and physical pollutants [2]. As the outermost barrier, skin is constantly exposed to a prooxidative environment (i.e., solar UVA and UVB radiation, air pollution), which can generate a large amount of reactive oxygen species [3,4,5]. The overproduction of these species can overwhelm the elaborated defense system of the human body, causing premature skin aging due to the induction of DNA and mitochondrial damage, lipid peroxidation, and activation of inflammatory signaling pathways. In addition, oxidative stress might negatively affect the natural healing process of skin lesions, increasing the risk of developing complications including infections, sepsis, or chronic wounds [6,7].

The daily use of natural antioxidants applied topically represents a valuable strategy aimed at protecting the skin from external insults and promoting the regeneration and healing of damaged tissues [8,9]. Due to increased interest in natural medicine, a large number of plant-derived antioxidants have been tested as potential bioactives for skin protection and repair. In vitro results obtained until now are encouraging, even if their effectiveness in vivo still has to be confirmed [10,11,12,13]. The use of innovative skin delivery systems, such as penetration enhancers or nanocarriers, represents a modern and valuable approach to maximize the local bioavailability of natural antioxidants [14,15,16]. Recent studies underlined the key role played by ad hoc formulated nanocarriers in improving the effectiveness of natural antioxidants in in vivo models of skin diseases [17,18,19].

Considering these challenges, in the present study, the extract of *Hypericum scruglii* Bacch., Brullo, and Salmeri (Hypericaceae) was loaded in ad hoc formulated phospholipid vesicles. *H. scruglii* is an endangered endemic plant species of the Sardinia Island [20] and it is considered as a plant of high pharmaceutical and conservation value [21,22]. In addition, these species are known for their ability to produce a wide spectrum of secondary metabolites, such as naphthodianthrones (hypericin and pseudohypericin), phloroglucinols (hyperforin and adhyperforin), phenolic acids, flavonoids (hyperoside, rutin, or quercitrin), xanthones, and terpenes [23]. In popular medicine, the oleolite of *Hypericum*, also called “St. John’s Wort Oil” and obtained by maceration of *H. perforatum* L. flowers in a vegetable oil, is well-known for its beneficial properties in skin diseases. It is traditionally used to treat burns, wounds, bedsores, and myalgias due to the high content of valuable components with antibacterial, antiviral, anti-inflammatory, astringent, and restoring properties [21,24]. Plant species belonging to the genus *Hypericum* has been used since ancient times for the treatment of different diseases and, recently, scientific researchers focused their attention on the phytochemicals contained in these plants, confirming the high content in polyphenols [25,26,27]. In addition, the extract of *H. scruglii* disclosed excellent antioxidant activity and inhibitory effects on elastase. The latter is a protease of the chymotrypsin family, responsible for the rupture of elastin as well as collagen and fibronectin [28].

The phytochemical composition and biological properties of *H. scruglii* have not been previously studied, but Mandrone et al. [23] have recently identified in this plant the presence of shikimic and chlorogenic acids, two derivatives of phloroglucinols, quercitrin, hyperoside, and hypericin, confirming their chemotaxonomic meaning. They also described the ability of these metabolites to exert antioxidant activity and inhibition of α-glucosidase.

In the present study, a hydro-alcoholic extract of the aerial parts of *H. scruglii* was obtained by a maceration process and its main components have been identified. The dried extract was loaded in ad hoc formulated glycerosomes aiming at improving its effectiveness. Glycerosomes were further modified by adding a natural dextrin (glucidex) and a natural polymer (gelatin or hyaluronan). Corresponding liposomes were prepared as well and used as reference. Vesicles were fully characterized and their biocompatibility was evaluated along with their ability to protect keratinocytes from oxidative stress and promote the closure of skin lesions. Finally, the uptake of vesicles by cells was assessed by using a confocal microscope.

## 2. Materials and Methods

### 2.1. Materials

Plants of *H. scruglii* were collected from the natural population of Funtanamela (Laconi, Italy) in July 2019. To ensure correct identification of the plant samples, the collected specimens were compared with the herbarium material harvested by Bacchetta at al. [20] in the same locality and preserved in the Herbarium CAG (University of Cagliari, Italy).

Lipids S75 (S75), a mixture of phospholipids (~70% soy phosphatidylcholine, 9% phosphatidylethanolamine, and 3% lysophosphatidylcholine), triglycerides, and fatty acids were purchased from AVG S.r.l. (Garbagnate Milanese, Milan, Italy), a local supplier for Lipoid GmbH (Ludwigshafen, Germany). Ethanol, glycerol, gelatin, 1,2-dioleolyl-snglycero-3-phosphoethanolamine-N-lissamine-sulfo-rhodamineB, 5(6)-carboxyfluorescein, Hoechst 33342, and all other reagents and solvents of analytical grade were purchased from Sigma-Aldrich (Milan, Italy). Analytical standards of fatty acids were purchased from Supelco Analytical (Bellefonte, PA, USA). Standard cyanidin-3-O-glucoside, petunidin-3-O-glucoside, peonidin-3-O-glucoside, malvidin 3-O-glucoside, delfinidin-3-O-glucoside, catechins, epicatechins, and gallic acid were purchased from Extrasynthese (Lyon, France). 2,2-diphenyl-1-pikryl-hydrazyl (DPPH) and Trolox were purchased from Sigma-Aldrich (St-Quentin Fallavier, France). Folin–Ciocalteu and sodium carbonate were purchased from Sigma-Aldrich (Darmstadt, Germany). All reagents and plastics for cell cultures were purchased from Life Technologies Europe (Monza, Italy).

### 2.2. Extraction of Phytocomplexes

The aerial parts of the plant were cleaned, washed, and left to dry in the dark for 30 days at room temperature. The dried aerial parts (100 g) were grinded to favor the extraction of the active components and then, dispersed in a mixture (1 L) of water and ethanol (30:70 *v*/*v*) and left under constant stirring for 48 h at room temperature (25 °C). The obtained dispersion was centrifuged twice (30 min, 8000 rpm). The extract was separated from the coarse part (precipitate); the ethanol was removed by evaporation at 45 °C and low pressure using a rotary evaporator. The remaining water was eliminated by a freeze-drying process. The extract obtained was finally stored under vacuum and protected from light.

### 2.3. Determination of the Total Phenolic Content

The quantification of total polyphenols was established by means of the Folin–Ciocalteu method [29]. A total of 200 µL of the extract was added to 1 mL of a diluted Folin–Ciocalteu solution and 800 µL of sodium carbonate. Samples were heated at 60 °C for 10 min and then, cooled in the refrigerator for another 10 min. The optical density was finally measured at a wavelength of 750 nm. A calibration curve was also built by using gallic acid as reference. Results were expressed as milligrams of gallic acid equivalent per gram of dry matter (mg GAE/g DM).

### 2.4. Estimation of the Antiradical Properties of the Extracts

The antiradical capacity of the extract was assessed by means of a DPPH (2,2-diphenyl-1-pikryl-hydrazyl) colorimetric method. A total of 50 µL of the extract was added to 1.45 mL of the DPPH (0.06 mM) solution and after incubation (30 min), the optical density was measured at 515 nm [30]. The calibration curve was obtained by using Trolox (positive control) and results were expressed as micrograms of Trolox equivalent per milliliter (µg TE/mL).

### 2.5. Polyphenol Characterization and Quantification by High Performance Liquid Chromatography (HPLC)

HPLC analyses were carried out to identify and quantify the polyphenols contained in the extracts, using an HPLC–DAD (diode array detection) (Waters Alliance, Milford, MA, USA), a quaternary Waters e2695 pump, a UV–vis photodiode array spectrophotometer (Waters 2998), a control system, and Empower 3 data collection software. HPLC studies were performed by using a Discovery HS C18, 5 μm, 250 × 4.6 mm, column with a HS C18, Supelguard Discovery, 20 × 4 mm, 5 μm, precolumn maintained at 30 °C. Two mobile phases were used during the analyses: mobile phase A, containing formic acid 0.2% (*v*/*v*) in water; mobile phase B, composed of a mixture of methanol (69%), water (29%), and formic acid (0.2%) (*v/v/v*). The HPLC gradient scheme was set as follows: 0% at 3 min, 10% at 10 min, 40% at 60 min, 60% at 80 min, 80% at 105 min, 100% at 120 min and again, 0% at 140 min, followed by 20 min of stabilization at 0%. Detection was assessed by changing the wavelengths from 278 to 364 nm, aiming at detecting all the bioactives of interest. A standard curve was determined for each compound (quercetin and chlorogenic acid) in a concentration range from 1 to 0.0625 mg/mL. The five standard solutions were made by progressive dilutions with a factor of 2. The linearity evaluation showed high correlation coefficients R^2^ (>99%). The detection of peaks was based on the standard’s retention time and comparison of the spectra. The limit of detection (LOD) and the limit of quantitation (LOQ) were evaluated by using the signal to noise ratio of chromatograms for blank samples: S/N = 3 and S/N = 10, respectively. The LOD of quercetin was 0.00013 mg/mL and the LOQ was 0.0005 mg/mL at 364 nm; likewise, for chlorogenic acid, the LOD was 0.00024 mg/mL and the LOQ was 0.0008 mg/mL at 324.8 nm [31,32,33].

### 2.6. Vesicle Preparation

Phospholipid S75 (240 mg) and *H. scruglii* extract (20 mg) were weighed in a glass vial and hydrated with 2 mL of water to obtain liposomes (used as reference) or 2 mL of a mixture of glycerol and water (25:75 *v*/*v*) to obtain glycerosomes. Glycerosomes were further modified by adding, to the mixture of phospholipid and extract, a commercial dextrin (glucidex) to obtain gluglycerosomes or the combination of glucidex and one polymer (gelatin or hyaluronan) to obtain gel-gluglycerosomes and hyal-gluglycerosomes, respectively. Dispersions were sonicated (40 cycles, 5 sec on and 2 sec off) with a Soniprep 150 ultrasonic disintegrator (MSE Crowley, London, UK) to obtain small vesicles homogeneously dispersed [34]. The direct sonication of dispersion is a rapid and green method as the use of organic solvents and dissipative processes is completely avoided. The composition of the formulations is reported in Table 1. Empty vesicles (i.e., without extract) were also prepared and characterized.

Vesicle dispersions were separated from the non-entrapped bioactives contained in the extract by dialysis against water (for liposomes) or the appropriate water and glycerol mixture (for glycerosomes, gluglycerosomes, gel-gluglycerosomes, and hyal-gluglycerosomes). Dispersions (1 mL) were loaded into dialysis tubes, Spectra/Por^®^ membranes (12–14 kDa MW cut-off, 3 nm pore size; Spectrum Laboratories Inc., DG Breda, The Netherlands), transferred in the dialyzing medium (3 L), and dialyzed for 2 h under continuous stirring at 25 °C. The medium was refreshed after 1 h. The entrapment efficiency was calculated as the percentage of the antioxidant activity of dispersion after dialysis versus that found before dialysis [35]. The antioxidant activity of vesicle dispersions was calculated by measuring their ability to scavenge the DPPH radical. Samples (20 µL) were dissolved in 1980 µL of DPPH methanolic solution (40 µg/mL) and incubated for 30 min at room temperature in the dark. At the end of the experiment, the absorbance (ABS) was measured at 517 nm against the blank. All the experiments were performed in triplicate. The antioxidant activity (AA%) was calculated as follows:

AA% = [(ABS_DPPH_ − ABS_sample_)/ABS_DPPH_] × 100

### 2.7. Evaluation of Physico-chemical Properties and Stability on Storage of Vesicles

The cryogenic transmission electron microscopy (cryo-TEM) method has been used to evaluate both the formation and morphology of vesicles. Specific grids covered with a holey carbon film were used to prepare the samples, which were immediately moved into an automatic plunge freezing apparatus (Vitrobot, FEI, Eindhoven, The Netherlands) aimed at controlling humidity and temperature. The formed film was then vitrified (Vitrobot, FEI Company, Eindhoven, The Netherlands) and observed by using a Tecnai F20 microscope (FEI Company) at −173 °C and 200 kV. Images were acquired by using a CCD Eagle camera (FEI Company).

Dynamic Light Scattering technique has been used to measure the average diameter and polydispersity index of vesicles by using a Zetasizer Ultra (Malvern Panalytical, Worcestershire, UK). The same Zetasizer Ultra was used to measure the zeta potential of samples, by means of the M3-PALS method. Water or the same mixture of water and glycerol used to prepare the vesicles have been used to dilute the sample (1:100), which was then analyzed at 25 °C.

A stability study was performed by measuring the size and size distribution of the vesicles stored at room temperature (25 °C) for 60 days.

### 2.8. Release Studies

The amount of extract released from the vesicles was measured by using a dissolution tester equipped with 6 stations (DT 720 Series—Erweka, distributed by EMME 3 SRL, Milan) according to USP requirements. Vesicle dispersions were transferred into polycarbonate dialysis tubes (Spectra/Por membranes: 12–14 kDa MW cut-off, 3 nm pore size; Spectrum Laboratories Inc., NJ, USA), put in the baskets of the dissolution tester containing the release media (1 liter), and left under constant stirring at 37 °C for 24 h. The amount of the extract in dispersion was evaluated at 2, 4, 6, 8, and 24 h by measuring the antioxidant activity using the DPPH colorimetric test.

### 2.9. Cell Viability and Protection against Oxidative Stress

Dulbecco’s Modified Eagle Medium (DMEM) with high glucose, supplemented with fetal bovine serum (10%), penicillin (100 U/mL), and streptomycin (100 µg/mL) has been used as a medium for the growth of immortalized human keratinocytes (HaCaT). Cells were maintained at 37 °C, 100% humidity, and 5% CO_2_ in 75 cm^2^ flasks. For the experiment, 7.5 × 10^3^ cells were seeded in each well of a 96-well plate and after 24 h, were treated with *H. scruglii* extract in aqueous dispersion (prepared by dispersing in water the same amount of extract used in the vesicles and sonicating it to obtain a more homogeneous dispersion) or loaded in vesicles, at different concentrations of the extract (20, 2, 0.2, and 0.02 g/mL). After 48 h, 100 µl of MTT 3-(4 5-dimethylthiazol yl-2)-2 5-diphenyltetrazolium bromide) reagent (0.5 mg/mL final concentration) was added to each well and plates were incubated for 3 h; then, formazan crystals produced by alive cells were dissolved by using dimethyl sulfoxide (100 µL), and the absorbance measured at 570 nm by using a microplate reader (Synergy 4 Reader, BioTek Instruments, AHSI S.p.A, Bernareggio, Italy). Experiments were performed three times, each time in triplicate. The cell viability of treated cells is shown as a percentage of untreated control cells (100% viability).

The protection of cells against the oxidative stress induced in keratinocytes with hydrogen peroxide has been evaluated by seeding 7.5 × 10^3^ cells/well into 96-well plates and once they reached semi-confluence, treating them with hydrogen peroxide in PBS (1:40,000) and simultaneously with *H. scruglii* extract in aqueous dispersion (prepared as reported above) or loaded in vesicles (2 and 0.2 µg/mL of *H. scruglii* extract). Cells were incubated for 4 h and two different controls were used: untreated cells (negative control) and cells treated with hydrogen peroxide only (positive control). After 4 h of incubation, cells were washed with fresh medium, and the MTT assay was used to measure the viability, as reported above. Results are reported as the percentage of untreated cells (100% viability).

### 2.10. Ability of H. Scruglii Extract Loaded into Vesicles to Promote Cell Proliferation and Migration: Scratch Assay

Keratinocytes were grown until complete confluence was reached. A linear scratch was generated in the cell monolayer by using a sterile pipette tip and the scattered fragments were accurately removed. The wounded area was then treated with *H. scruglii* extract in dispersion or loaded into vesicles (0.2 µg/mL of *H. scruglii* extract) and monitored by using an optical microscope (10× objective), at scheduled time intervals (0, 12, 24, 36, and 48 h). The observation result allowed the evaluation of the ability of the different formulations to stimulate proliferation and migration of keratinocytes.

Images at time zero (t = 0 h) were captured to record the initial area of the wounds, and the recovery of the wounded monolayers due to cell migration and proliferation was evaluated at 12, 24, 36, and 48 h (t = Δ h). The captured images were quantified by Java’s image J software (1.8.0_172, http://rsb.info.nih.gov) by measuring the area of the wound [36]. The migration of cells toward the wounds was expressed as percentage of wound closure:

% of wound closure = [(a (0 h) − a (∆ h)/a (0 h)] × 100%

where a (0 h) is the wounded area immediately after scratching, and a(Δ h) is the wounded area measured at 12, 24, 36, and 48 h after scratching.

### 2.11. Uptake of Fluorescent Vesicles by Keratinocytes

Fluorescent vesicles, labelled with a lipophilic fluorescent marker (1,2-dioleolyl-snglycero-3-phosphoethanolamine-N-lissamine-sulfo-rhodamineB; Rho-PE, 0.025 mg/mL; red) and loaded with a hydrophilic fluorescent marker (5(6)-carboxyfluorescein; CF, 0.025 mg/mL; green), were prepared to evaluate their internalization by keratinocytes [37]. Poly-L-Lysine-coated 8-well μ-slides (Ibidi GmbH, Martinsried, Munich, Germany) were used to culture the keratinocytes, which were then treated with the vesicular formulations for 2, 4, and 24 h. At each time point, alive cells were stained with the markers for the cell nucleus (Hoechst 33342, Trihydrochloride, Trihydrate −10 mg/mL solution in water; blue) and observed by using a confocal inverted microscope FluoView FV1000 (Olympus, Barcelona, Spain) equipped with a UV–visible light laser and a 60× objective UPLSAPO. Fluorescence excitation and emission wavelengths for RhoPE, CF, and Hoechst were 559/578, 470/535, and 360/460 nm, respectively.

### 2.12. Statistical Analysis of Data

The results were expressed as mean value ± standard deviation. Statistically significant differences among samples were determined by using variance analysis. The post hoc Tukey–Kramer *t*-test was used to substantiate a significant difference between the means of two specific groups. The statistical analysis was performed by using the Excel software package (Microsoft Corp, Redmond, WA, USA) equipped with a tool for statistical analysis. The minimum level of significance chosen was *p* < 0.05.

## 3. Results

### 3.1. Extract Characterization

**Figure 1:**
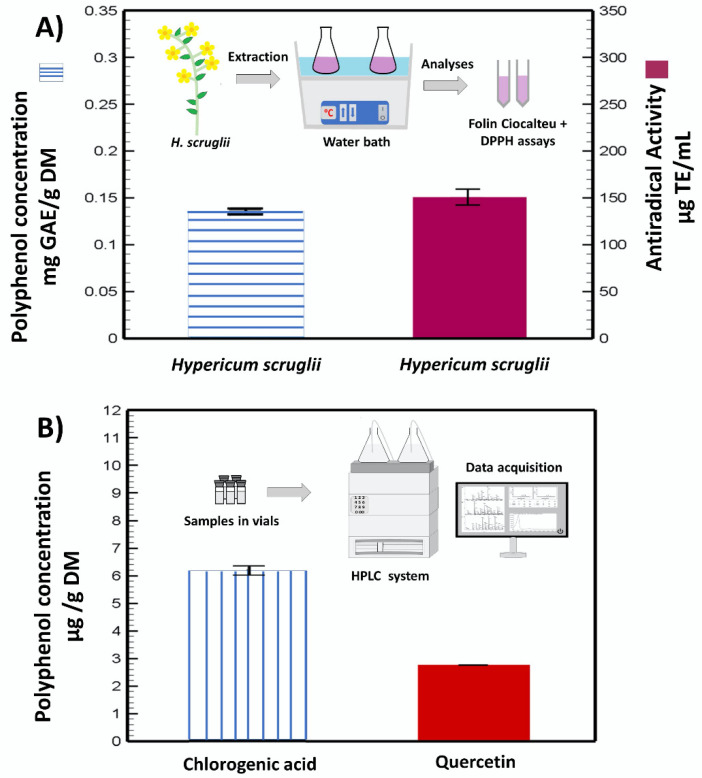
Polyphenol concentration and antiradical activity of the *H. scruglii* extract (inset shows the schematic representation of the process) (**A**), and chlorogenic and quercetin concentrations identified by HPLC (inset shows the schematic representation of the HPLC method) (**B**).

The extraction method is schematically represented in Figure 1A, along with the assayed total phenolic content and the radical scavenging capacity of *H. scruglii* extract. The extract contained 0.13 ± 0.0018 mg GAE/g DM and exhibited an antiradical capacity of 150.4 ± 9.9 µg TE/mL.

Two main molecules were identified and quantified in the *H. scruglii* extract by HPLC analyses: quercetin (flavonoid) and chlorogenic acid (phenolic acid). The amount of chlorogenic acid (~6 µg/g DM) was almost double that of quercetin (~3 µg/g DM) (Figure 1B).

### 3.2. Vesicle Characterization

Glycerosomes were used as skin delivery systems for the extract obtained from the areal parts of *H. scruglii*. The performances of glycerosomes were further improved by adding a natural dextrin (glucidex) to prepare gluglycerosomes and one polymer (gelatin or hyaluronan), thus obtaining gel-gluglycerosomes and hyal-gluglycerosomes.

**Figure 2:**
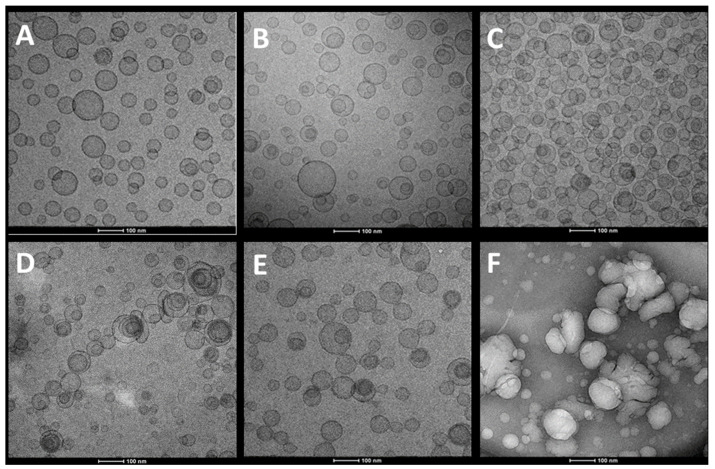
Representative cryo-TEM images of liposomes (**A**), glycerosomes (**B**,**C**), gluglycerosomes (**D**), gel-gluglycerosomes (**E**), and hyal-gluglycerosomes (**F**), loading *H. scruglii* extract.

Cryo-TEM analyses disclosed that *H. scruglii* extract-loaded liposomes, used as reference, were spherical and unilamellar (Figure 2A). Glycerosomes (Figure 2B,C) and gluglycerosomes (Figure 2D) assembled in spherical and oligolamellar vesicles, which appeared close-packed. The addition of gelatin did not significantly modify both morphology and structure of the glycerosomes (Figure 2E), while the addition of hyaluronan (hyal-gluglycerosomes) allowed the formation of aggregated vesicles with an irregular shape (Figure 2F). The results underlined that the glycerol facilitated the formation of concentric bilayer probably because it decreased the repulsion between the bilayer surfaces. Dextrin and gelatin did not affect the assembly probably because of their possible location in the water phase, within the lipid bilayers, and in the intervesicle’s medium [38]. Hyaluronan seems to facilitate the aggregation of vesicles.

The mean diameter of the vesicles was measured by means of dynamic laser light scattering technique, which provided a global estimation of the vesicle size calculated from the scattering intensity of each particle fraction (Table 2). Extract-loaded liposomes and empty vesicles were prepared and characterized as well. The mean diameter of empty vesicles was ~116 nm without statistical differences among species (*p* > 0.05). The loading of the extract did not significantly affect the size of liposomes and glycerosomes, which have a mean diameter ~120 nm. On the contrary, the loading of the extract caused an increase in the size of gluglycerosomes with or without gelatin or hyaluronan, which disclosed the same mean diameter, ~158 nm (*p* > 0.05 among the three formulations of extract-loaded gluglycerosomes), according to the results reported by Akgün et al. [39] for chitosan-coated liposomes. Indeed, for these formulations, the empty vesicles were smaller than the corresponding extract-loaded vesicles, indicating a contribution of the extract in both assembly and curvature radius of the bilayer. As previously reported, dextrin may stabilize the system thanks to their localization in both inter-lamellar and inter-vesicle medium [40]. In particular, its distribution in the aqueous compartment of the vesicles combined with the active component of the extract may cause a reduced curvature radius of vesicles, thus leading to the formation of bigger systems [41]. All the dispersions of *H. scruglii* extract-loaded vesicles were more homogeneously dispersed (polydispersity index ≤ 0.30) in comparison with empty vesicles (polydispersity index ~0.34), probably because the components of the extract may affect the vesicle assembly leading to the formation of vesicles more similar in size, as reported in previous studies where polyphenols influenced phospholipid assembly and reduced aggregation phenomena [42,43]. The zeta potential was highly negative for all the extract-loaded vesicles (~−48 mV, *p* > 0.05 among all) irrespective of their composition. The results are in agreement with other studies, which confirmed the negative charge of liposomes made with the zwitterionic phosphatidylcholine [39,44]. The amount of extract incorporated into the vesicles was very high (entrapment efficiency ~89%) without significant differences among the different samples. As expected, the antioxidant activity (AA, Table 2) of vesicles was very high and the composition of vesicles did not modify the antioxidant power of the extract, as already reported [38,45].

**Figure 3:**
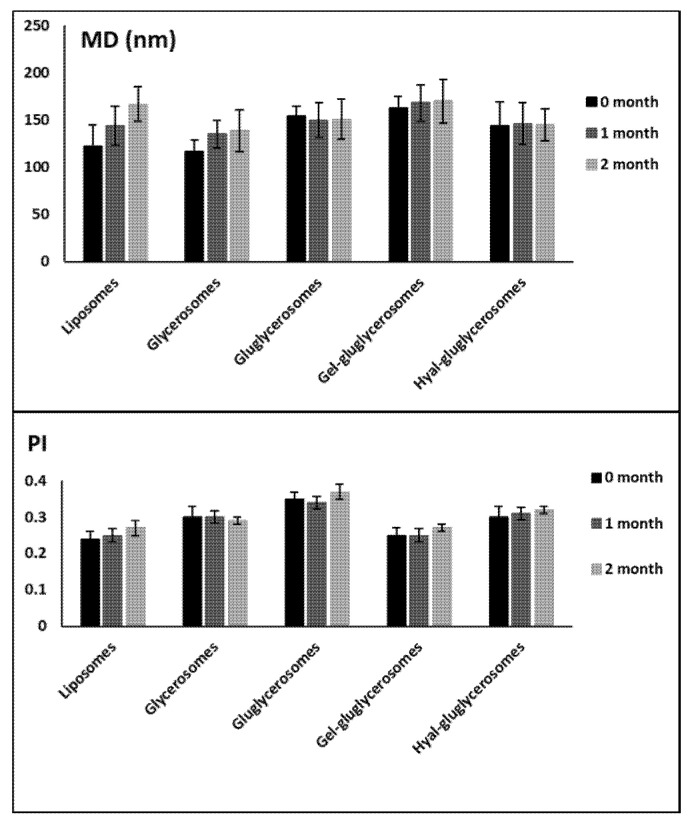
Mean diameter and polydispersity index (PI) of vesicles containing *H. scruglii* extract over 60 days of storage at room temperature (25 °C). Mean values ± standard deviations are reported (n = 6).

Stability studies performed for 60 days at room temperature underlined the positive effect of the combination of dextrin and polymers on this property. Indeed, any significant variation of size and polydispersity index has been detected for these formulations, while an increase in vesicle size was detected for glycerosomes and liposomes, used as reference (Figure 3).

### 3.3. Release Studies

**Figure 4:**
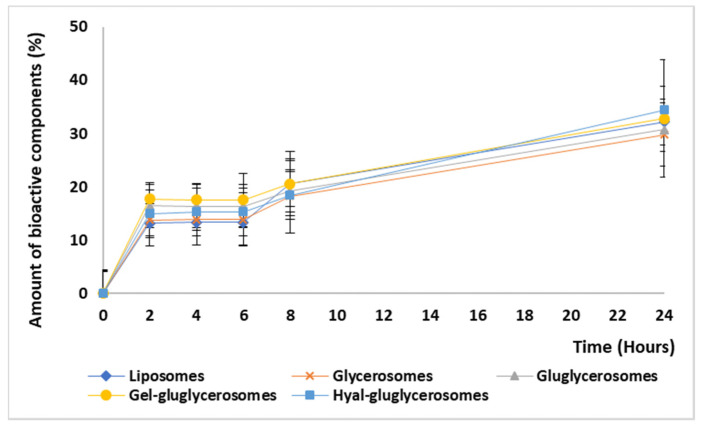
Amount of bioactive components (%) released from liposomes, glycerosomes, gluglycerosomes, gel-gluglycerosomes, and hyal-gluglycerosomes during 24 h of experiment. Mean values (error bars) ± standard deviations are reported (n = 3).

The amount of extract released by the vesicles during 24 h was similar for all the used vesicles. A burst release was observed at 2 h, reaching 20% of the initial amount; after, it became almost constant up to 24 h, reaching ~35%, Figure 4. Any significant differences between the formulations tested have been detected, suggesting that the bioactives contained in the extract are effectively incorporated in the vesicles. The slow release of the bioactives can be exploited at the skin level to reach an improvement in their effect as the formulation can stay at the application site as a depot, slowly releasing the actives in the damaged area and favoring the interaction with cells [46,47].

### 3.4. Biocompatibility and Protective Effect against Oxidative Stress of H. scruglii Extract-Loaded Vesicles

**Figure 5:**
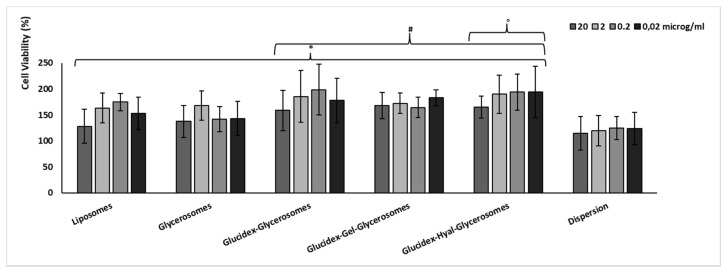
Viability of keratinocytes incubated for 48 h with different concentrations of *H. scruglii* extract in dispersion or loaded in vesicles. Data are reported as mean values ± standard deviations of cell viability expressed as the percentage of control (untreated cells; 100% of viability). The symbol (*) indicates values statistically different from dispersion (*p* < 0.05); (#) indicates values statistically different from glycerosomes (*p* < 0.05); (°) indicates values statistically different from liposomes (*p* < 0.05).

The biocompatibility of the *H. scruglii* extract in aqueous dispersion or loaded in vesicles was evaluated by using keratinocytes, which are the outermost cells of the skin. No toxic effect was observed in cells treated with either vesicles or dispersion of the extract (Figure 5). Furthermore, cell proliferation was detected especially using extract-loaded vesicles, which improved the cell viability up to ≥150%, without significant differences among samples and concentrations (*p* > 0.05). The proliferative effect was less evident when cells were treated with the extract in dispersion as the viability was ~120% irrespective of the concentration used. The higher viability provided by treating the cells with the extract-loaded vesicles can be related to their ability to interact with cells favoring the internalization of the active components of the extract [48,49].

**Figure 6:**
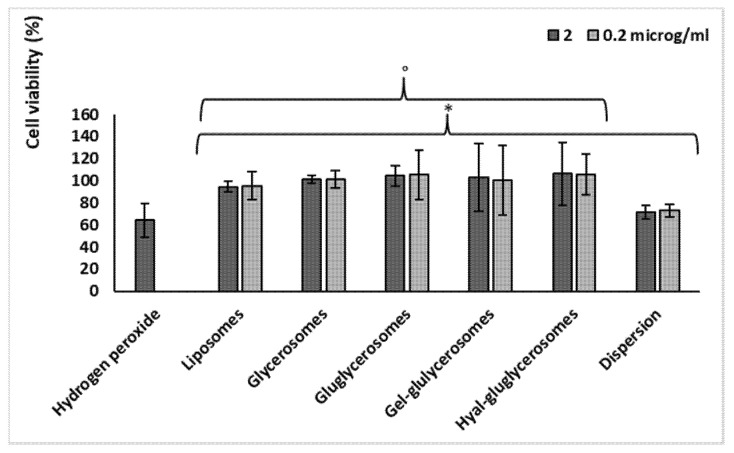
Viability of keratinocytes stressed with hydrogen peroxide and treated with *H. scruglii* extract in dispersion or loaded in vesicles. Data (bars) are reported as mean values ± standard deviations of cell viability expressed as the percentage of control (untreated cells; 100% of viability). The symbol (*) indicates values statistically different from that obtained with hydrogen peroxide (*p* < 0.05); (°) indicates values statistically different from that obtained with dispersion (*p* < 0.05).

Likewise, *H. scruglii* extract was able to protect keratinocytes against damages induced by hydrogen peroxide, which was chosen as the oxidative agent (Figure 6). Its loading into vesicles strengthened its protective effect. Indeed, cells stressed with hydrogen peroxide showed a low viability (~62%), which did not change with the simultaneous treatment with extract in dispersion, irrespective of the used concentration (72%). When the extract was loaded in vesicles, the cell viability reached ~100% (*p* > 0.05 among cells treated with different vesicles, and *p* < 0.05 versus cells stressed with hydrogen peroxide and treated with the extract in dispersion). The treatment with the extract-loaded vesicles was able to counteract the damages induced by using hydrogen peroxide, re-establishing the healthy conditions. Therefore, the improved effectiveness of *H. scruglii* extract observed when it was incorporated in the vesicles may confirm our hypothesis according to which, thanks to their ability to interact with cells, vesicles may promote the release of the bioactives inside the cytoplasmic environment, leading the neutralization of the oxidative species. No differences in antioxidant power were detected among the different formulations.

### 3.5. Wound Healing Activity

**Figure 7:**
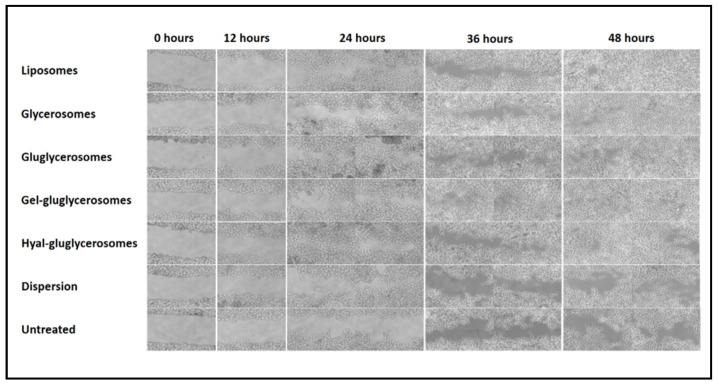
Representative optical microscopy images at different time points (0, 12, 24, 36, and 48 h) of the wound induced in keratinocytes and treated with *H. scruglii* extract in dispersion or loaded in vesicles. Bars correspond to 200 μm.

The promotion of both proliferation and migration of keratinocytes was monitored during the treatment of the wounded area with *H. scruglii* extract in dispersion or loaded in vesicles. As shown in Figure 7, *H. scruglii* extract-loaded vesicles promoted the closure of the wound to a greater extent than the extract in dispersion, used as reference.

The scratch of untreated keratinocytes was mostly unchanged at 24 h, the proliferation and migration of cells started at 36 h, and closure of the wound was not reached at 48 h. A similar behavior was observed in cells treated with *H. scruglii* extract in dispersion, even if the proliferation and migration of cells started at 24 h. The treatment with extract-loaded vesicles stimulated to a greater extent the proliferation and migration of cells, especially when gel-gluglycerosomes were used, as they lead to the almost complete closure of the scratch already at 36 h. After 48 h, the wound was almost completely closed for cells treated with all the other vesicles, confirming one more time the key contribution of the carriers in promoting the effectiveness of the extracts. To better evaluate the differences provided by the different formulations, the wounded areas were measured and the healing areas were reported as a function of the time (Figure 7).

**Figure 8:**
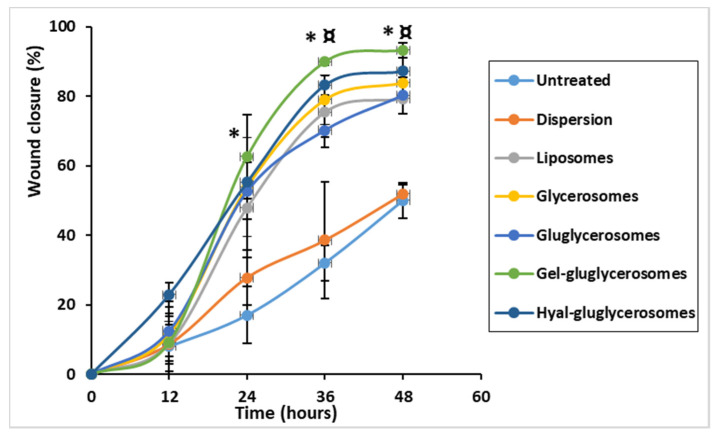
Percentage of wound closure. Wound healing is expressed as percentage of closure relative to the original size of wound. Mean values ± standard deviations are reported. The symbol (*) indicates values significantly different from that of untreated keratinocytes (control) and keratinocytes treated with the extract dispersion at 24, 36, and 48 h. The symbol (¤) indicates values significantly different from that of cells treated with gel-gluglycerosomes and other formulations.

The lesion of untreated cells was only partially closed (~50%) after 48 h, which gives an idea of the proliferation and migration speed of normal cells, mimicking the normal and healthy conditions (Figure 7 and Figure 8). Likewise, when the cells were treated with *H. scruglii* extract in dispersion, the closure of the wound at 48 h was ~50%. A faster closure of the wound was observed when the cells were treated with *H. scruglii* extract-loaded vesicles. Particularly using gel-gluglycerosomes, the % of closure reaches ~89% at 32 h and increased more at 48 h to reach ~93%. Using the other phospholipid vesicles, the closure of the wound reached ~76% at 32 h and ~83% at 48 h, which, compared with the closure of the wound treated with gel-gluglycerosomes, was significantly lower (* ¤; *p* < 0.05). This study confirmed the optimal performances of all the vesicles as they promoted the delivery of *H. scruglii* extract and favored the proliferation and migration of keratinocytes. Particularly, the gel-gluglycerosoemes were found to be the most suitable formulation for the treatment of the wound induced in a monolayer of keratinocytes.

### 3.6. Uptake of Fluorescently Labelled Vesicles by Keratinocytes

**Figure 9:**
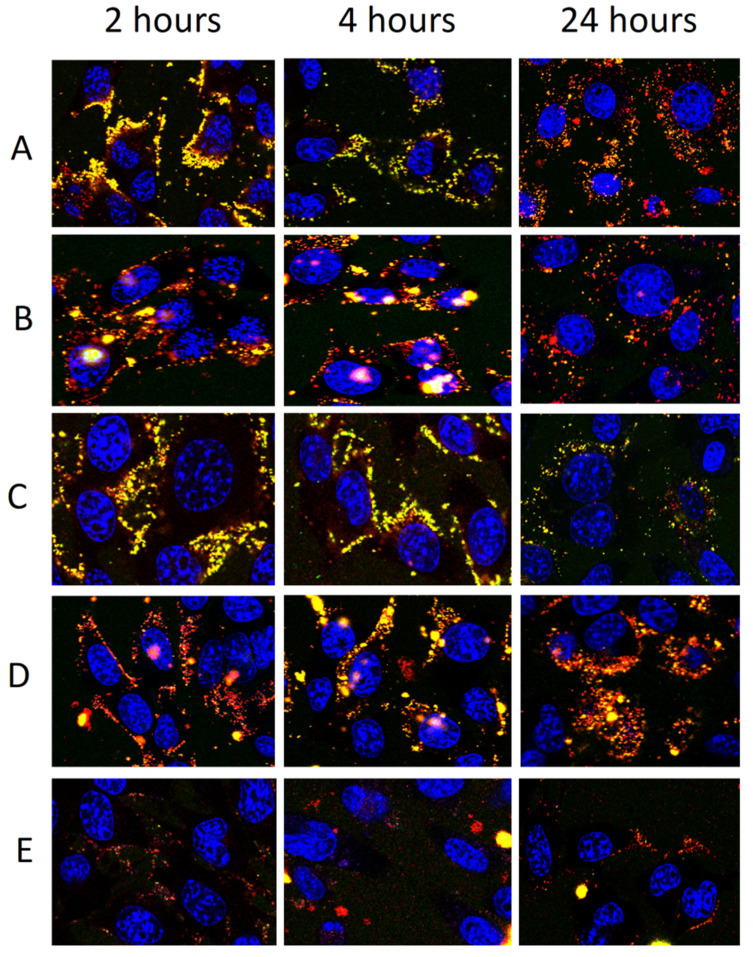
Confocal laser scanning microscopy images of living keratinocytes incubated for 24 h with fluorescently labelled vesicles: liposomes (**A**), glycerosomes (**B**), gluglycerosomes (**C**), gel-gluglycerosomes (**D**), and hyal-gluglycerosomes (**E**). The localization and intensity of the dyes are displayed at 2, 4, and 24 h in red for rhodamine-phosphoethanolamine, green for carboxyfluorescein, and blue for the nucleus. The orange area indicated the superposition of the markers (internalization of intact vesicles). Bars correspond to 50 μm.

To confirm our findings, the ability of vesicles to interact with cells and promote their internalization has been evaluated. Keratinocytes were treated with fluorescent vesicles, obtained by labelling them with a lipophilic fluorescent marker (Rho-PE, 0.025 mg/mL) and loading a hydrophilic fluorescent marker (CF, 0.025 mg/mL), for 2, 4, and 24 h. Treated cells were observed by using a confocal microscope to evaluate the trend of internalization at the different time points (Figure 9).

After 2 h of incubation, yellow fluorescence was evident around and inside the cells by using liposomes, disclosing a rapid internalization of vesicles. At 2 h of treatment with glycerosomes (with or without glucidex and gelatin), the main fluorescence of both probes was less evident and located around the cell membrane as a small point, probably formed by aggregates of vesicles. Fluorescence was not evident at each time point inside the cells when hyal-gluglycerosomes were used, probably because of the reduced uptake due to the interaction between hyaluronan and glucidex and the quick aggregation of vesicles as disclosed in cryo-TEM images. At 4 h, using liposomes, the fluorescence of both markers was feeble, punctuated and distributed on the cell membrane denoting a rapid elimination, which was even more evident at 24 h. Using glycerosomes, gluglycerosomes, and gel-gluglycerosomes, the uptake of probes in the cytoplasm of the cells was evident at 4 and 24 h, disclosing a delayed but more effective uptake of these formulations.

## 4. Discussion

Different species of *Hypericum* have been widely used in the treatment of skin wounds in European phytotherapy and Turkish folk medicine [50]. Mukherjee et al. [51,52] confirmed the effect of *H. perforatum* and *H. patulum* Thunb. extracts on in vivo lesions and Süntar et al. [53] corroborated the healing and anti-inflammatory activities of *H. perforatum*.

In this study, the extract of *H. scruglii* was obtained from the aerial parts and its main components were identified. The composition of the extract confirmed its high antioxidant activity and antiradical activities, which were detected by in vitro tests. According to this, previous studies reported that the antioxidant capacity of *Hypericum* species is mainly related to their flavonoids and phenolic acids content [54]. Mandrone et al. [23], by using H-NMR, identified chlorogenic acid and a quercetin derivative in the extract obtained from *H. perforatum*. Similarly to the extract of *H. scruglii* obtained from the aerial part, the one obtained from the leaves of *H. hircinum* L. contained chlorogenic acid and quercetin [55]. However, the extract obtained from *H. scruglii* was richer in chlorogenic acid than both *H. hircinum* and *H. perforatum* extracts. In addition, in this study, the wound healing ability of *H. scruglii* extract was confirmed and can be related to the presence of quercetin (flavonoid) and chlorogenic acid (phenolic acid). Indeed, the ABTS and DPPH assays showed that *H. scruglii* extract has higher antioxidant capacities than *H. perforatum* extracts [23]. Their antiradical activities seem to exert enzymatic inhibition and many polyphenols, especially flavonoids (i.e., resveratrol derivatives, ellagic acid, etc.), exhibited the inhibition of tyrosinase and elastase enzymes [56,57,58]. A positive correlation (Pearson test) was found between the total phenolic and flavonoid content of *H. scruglii* extracts and that of tyrosinase and elastase inhibition [28]. This effect is expected to increase the concentration of elastin and tyrosine, which are functional proteins and play a critical role in wound pathogenesis [59].

Accordingly, for the first time in this study, the effectiveness of the *H. scruglii* extract was demonstrated and improved by its loading in phospholipid vesicles. Indeed, in previous studies, only hypericin was loaded into the liposomes and different complexes were formulated for other aims [60,61], while different kinds of liposomes and phospholipid vesicles have been designed and tested, aimed at improving the efficacy of plant extracts [62,63]. The results underlined that the composition of the vesicles and the addition of specific additives can positively affect the effectiveness of the payloads, even if for each bioactive or phytocomplex, an ad hoc formulation is required [35,64,65]. Previous works strengthened the positive effect of glycerosomes on promoting the accumulation of bioactives in the different skin strata, mainly because of the moisturizing effect of glycerol, which is able to modify the ordered structure of the stratum corneum and favor the passage of bioactives [66]. They have been used to deliver several natural molecules, extracts, and oils in the skin, providing an enhancement of the local bioavailability of the payload in comparison with liposomes [67]. Results confirmed that glycerosomes are versatile vesicles that can: (i) improve the delivery of several kinds of molecules [67,68], (ii) be modified with one or more additives [69], and (iii) be used for different administration routes [70].

In light of these promising findings, in the present work, the extract of *H. scruglii* has been loaded in glycerosomes modified with specific additives (maltodextrin alone or combined with gelatin or hyaluronan), which are expected to improve the stability and the viscosity of the dispersions and their ability to deliver the payloads to the skin [71,72,73]. Glucidex, a maltodextrin with a dextrose equivalent of 17, is a water soluble dextrin and can act as a structural component, which is dispersed in the vesicle surface and, in the water phase between the bilayers, may exert a mechanical reinforcement, improving their stability and ensuring their cryopreservation [74,75]. In addition, this polysaccharide also seems to play a functional role in wound healing, especially in association with gelatin. Indeed, gel-gluglycerosomes provided the more rapid and effective closure of skin lesions in the scratch assay, probably because of the ability of glucidex and/or its combination with gelatin, to promote cell proliferation to a better extent than the other components of the vesicles. This result is in agreement with previous studies reporting the wound healing promotion of different polysaccharides [76,77]. The most promising efficacy of gel-gluglycerosomes can also be due to the delayed and prolonged uptake provided by all glycerosomes in comparison with liposomes. The extract can remain inside the cells for more time, favoring their proliferation and migrations.

The addition of the dextrin, alone or in combination with the polymer (gelatin or hyaluronan), slightly affects the size of the extract-loaded vesicles, allowing a low increase in their mean diameter. The increased size can be related to its deposition on the vesicle surface [78]. However, the presence of glucidex alone or in combination with gelatin or sodium hyaluronate improved the stability of the dispersions, probably because of the formation of a more viscous and structured medium capable of avoiding aggregation and fusion, as previously reported for polymer associated vesicles [38,79,80]. 

In vitro results performed by using keratinocytes, as the most representative cells of the human skin, underlined the high biocompatibility of all tested vesicles. All the vesicles, irrespective of composition, were able to improve the ability of the extract to protect the keratinocytes from the damages induced by hydrogen peroxide. Indeed, the extract-loaded vesicles protect the cells to a better extent than the dispersion, used as reference.

Compared to gelatin, the combination of hyaluronan and glucidex did not give promising results in the delivery of *H. scruglii* extract, demonstrating that for each extract, it is important to find the most suitable formulation. Indeed, even if sodium hyaluronate is well-known for its capacity of stimulating cell proliferation and migration, in this case, its effect is slightly reduced in comparison with that provided by gelatin, which, in combination with glucidex and glycerosomes, ensured the optimal performance of the vesicles. Gel-gluglycerosomes seemed to be the most promising formulations considering that they were the most effective in both counteracting the oxidative stress and promoting the healing of an induced wound in cells. All these findings confirmed the key role played by the vesicles, which interact with cells favoring the release of the payload inside the cytoplasm, where they can exert their beneficial effect.

## 5. Conclusions

This work underlines that *H. scruglii* extract is effective in the treatment of skin lesions related to oxidative stress and its loading into specific phospholipid vesicles improved its efficacy, especially when glycerosomes were used and modified with the combination of dextrin and gelatin. These vesicles addressed great antioxidant activity along with better cell uptake and wound healing effects. The results disclosed that *H. scruglii* extract-loaded gel-gluglycerosomes are promising carriers to be used in the treatment of skin lesions connected with oxidative processes due to external insults and lack of internal homeostasis and, in addition, confirmed that for each phytocomplex, it is necessary to find the most suitable formulation, capable of effectively ensuring the highest efficacy. Overall, the results are, however, not enough to confirm the effectiveness of *H. scruglii* extract incorporated into vesicles for the treatment of skin diseases and in vivo and clinical evaluation are needed to support in vitro results.

## Figures and Tables

**Table 1:** Composition of *Hypericum scruglii* extract-loaded vesicles.

	S75 (mg/mL)	Extract (mg/mL)	Glucidex (mg/mL)	Gelatin (mg/mL)	Hyaluronan (mg/mL)	Glycerol (mL)	Water (mL)
Liposomes	120	10	0	0	0	-	1
Glycerosomes	120	10	0	0	0	0.25	0.75
Gluglycerosomes	120	10	25	0	0	0.25	0.75
Gel-gluglycerosomes	120	10	25	1	0	0.25	0.75
Hyal-gluglycerosomes	120	10	25	0	1	0.25	0.75

**Table 2:** Mean diameter (MD), polydispersity index (PI), zeta potential (ZP), and entrapment efficiency (EE) of *H. scruglii* extract-loaded vesicles. Mean values ± standard deviations are reported (n = 6).

	DM (nm)		PI		ZP (mV)		EE (%)	AA (%)
	Empty	Extract	Empty	Extract	Empty	Extract	Extract	Extract
Liposomes	111 ± 13	122 ± 23	0.36	0.24	−39 ± 5	−55 ± 6	88 ± 9	64 ± 4
Glycerosomes	108 ± 19	117 ± 15	0.32	0.30	−41 ± 6	−53 ± 5	85 ± 7	79 ± 7
Gluglycerosomes	121 ± 21	154 ± 22	0.35	0.30	−37 ± 3	−41 ± 4	88 ± 11	84 ± 6
Gel-gluglycerosomes	114 ± 16	163 ± 33	0.34	0.25	−43 ± 7	−39 ± 6	97 ± 4	83 ± 7
Hyal-gluglycerosomes	127 ± 24	154 ± 25	0.35	0.29	−39 ± 4	−50 ± 5	88 ± 13	81 ± 9

## References

[1] Gefen A. (2019). Innovations and Emerging Technologies in Wound Care.

[2] Fore J. (2006). A review of skin and the effects of aging on skin structure and function. Ostomy/Wound Manag..

[3] Birch-Machin M.A., Russell E.V., Latimer J.A. (2013). Mitochondrial DNA damage as a biomarker for ultraviolet radiation exposure and oxidative stress. Br. J. Dermatol..

[4] Packer L., Valacchi G. (2002). Antioxidants and the Response of Skin to Oxidative Stress: Vitamin E as a Key Indicator. Ski. Pharmacol. Physiol..

[5] Birch-machin M.A., Swalwell H. (2010). How mitochondria record the effects of UV exposure and oxidative stress using human skin as a model tissue. Mutagenesis.

[6] Church D., Elsayed S., Reid O., Winston B., Lindsay R. (2006). Burn Wound Infections. Clin. Microbiol. Rev..

[7] Godbout J.P., Glaser R. (2006). Stress-Induced Immune Dysregulation: Implications for Wound Healing, Infectious Disease and Cancer. J. Neuroimmune Pharmacol..

[8] Briganti S., Picardo M. (2003). Antioxidant activity, lipid peroxidation and skin diseases. What’s new. J. Eur. Acad. Dermatol. Venereol..

[9] Tabassum N., Hamdani M. (2014). Plants used to treat skin diseases. Pharm. Rev..

[10] Halliwell B. (2008). Are polyphenols antioxidants or pro-oxidants? What do we learn from cell culture and in vivo studies?. Arch. Biochem. Biophys..

[11] Chua L.S., Lee S.Y., Abdullah N., Sarmidi M.R. (2012). Review on *Labisia pumila* (Kacip Fatimah): Bioactive phytochemicals and skin collagen synthesis promoting herb. Fitoterapia.

[12] Nicolaou A. (2013). Eicosanoids in skin inflammation. Prostaglandins Leukot. Essent. Fat. Acids.

[13] De Luca C., Mikhal’chik E.V., Suprun M.V., Papacharalambous M., Truhanov A.I., Korkina L.G. (2016). Skin Antiageing and Systemic Redox Effects of Supplementation with Marine Collagen Peptides and Plant-Derived Antioxidants: A Single-Blind Case-Control Clinical Study. Oxid. Med. Cell. Longev..

[14] Sala M., Diab R., Elaissari A., Fessi H. (2018). Lipid nanocarriers as skin drug delivery systems: Properties, mechanisms of skin interactions and medical applications. Int. J. Pharm..

[15] Zhang Z., Tsai P., Ramezanli T., Michniak-Kohn B.B. (2013). Polymeric nanoparticles-based topical delivery systems for the treatment of dermatological diseases. WIREs Nanomed. Nanobiotechnol..

[16] Cristiano M.C., Froiio F., Spaccapelo R., Mancuso A., Nisticò S.P., Udongo B.P., Fresta M., Paolino D. (2020). Sulforaphane-loaded ultradeformable vesicles as a potential natural nanomedicine for the treatment of skin cancer diseases. Pharmaceutics.

[17] Coradini K., Lima F.O., Oliveira C.M., Chaves P.S., Athayde M.L., Carvalho L.M., Beck R.C.R. (2014). Co-encapsulation of resveratrol and curcumin in lipid-core nanocapsules improves their in vitro antioxidant effects. Eur. J. Pharm. Biopharm..

[18] Scalia S., Franceschinis E., Bertelli D., Iannuccelli V. (2013). Comparative Evaluation of the Effect of Permeation Enhancers, Lipid Nanoparticles and Colloidal Silica on in vivo Human Skin Penetration of Quercetin. Ski. Pharmacol. Physiol..

[19] Pivetta T.P., Simões S., Araújo M.M., Carvalho T., Arruda C., Marcato P.D. (2018). Development of nanoparticles from natural lipids for topical delivery of thymol: Investigation of its anti-inflammatory properties. Colloids Surf. B Biointerfaces.

[20] Bacchetta G., Brullo S., Salmeri C. (2010). *Hypericum scruglii* sp. nov.(Guttiferae) from Sardinia. Nord. J. Bot..

[21] Sanna C., Scognamiglio M., Fiorentino A., Corona A., Graziani V., Caredda A., Cortis P., Montisci M., Ceresola R., Canducci F. (2018). Prenylated phloroglucinols from *Hypericum scruglii*, an endemic species of Sardinia (Italy), as new dual HIV-1 inhibitors effective on HIV-1 replication. PLoS ONE.

[22] Porceddu M., Sanna M., Serra S., Manconi M., Bacchetta G. (2020). Seed germination requirements of *Hypericum scruglii*, an endangered medicinal plant species of Sardinia (Italy). Botany.

[23] Mandrone M., Scognamiglio M., Fiorentino A., Sanna C., Cornioli L., Antognoni F., Bonvicini F., Poli F. (2017). Phytochemical profile and α-glucosidase inhibitory activity of Sardinian *Hypericum scruglii* and *Hypericum hircinum*. Fitoterapia.

[24] Stojanovic G., Dordevic A., Smelcerovic A. (2013). Do other *Hypericum* species have medical potential as St. John’s wort (*Hypericum perforatum*)?. Curr. Med. Chem..

[25] Saddiqe Z., Naeem I., Maimoona A. (2010). A review of the antibacterial activity of *Hypericum perforatum* L.. J. Ethnopharmacol..

[26] Zhang X.-W., Ye Y.-S., Xia F., Yang X.-W., Xu G. (2019). Diverse Polyphenols from *Hypericum faberi*. Nat. Prod. Bioprospecting.

[27] Zhang R., Ji Y., Zhang X., Kennelly E.J., Long C. (2020). Ethnopharmacology of *Hypericum* species in China: A comprehensive review on ethnobotany, phytochemistry and pharmacology. J. Ethnopharmacol..

[28] Chiocchio I., Mandrone M., Sanna C., Maxia A., Tacchini M., Poli F. (2018). Screening of a hundred plant extracts as tyrosinase and elastase inhibitors, two enzymatic targets of cosmetic interest. Ind. Crops Prod..

[29] Libbey L.M., Walradt J.P. (1968). 3,5-di-Tert-butyl-4-hydroxytoluene (BHT) as an artifact from diethyl ether. Lipids.

[30] Kallithraka S., Mohdaly A.A.A., Makris D.P., Kefalas P. (2005). Determination of major anthocyanin pigments in Hellenic native grape varieties (*Vitis vinifera* sp.): Association with antiradical activity. J. Food Compos. Anal..

[31] Taamalli A., Arráez-Román D., Barrajón-Catalán E., Ruiz-Torres V., Segura-Carretero A., Fernández-Gutiérrez A. (2012). Use of advanced techniques for the extraction of phenolic compounds from Tunisian olive leaves: Phenolic composition and cytotoxicity against human breast cancer cells. Food Chem. Toxicol..

[32] Rajha H.N., Abi-Khattar A.M., El Kantar S., Boussetta N., Lebovka N., Maroun R.G., Louka N., Vorobiev E. (2019). Comparison of aqueous extraction efficiency and biological activities of polyphenols from pomegranate peels assisted by infrared, ultrasound, pulsed electric fields and high-voltage electrical discharges. Innov. Food Sci. Emerg. Technol..

[33] Rajha H.N., Mhanna T., El Kantar S., El Khoury A., Louka N., Maroun R.G. (2019). Innovative process of polyphenol recovery from pomegranate peels by combining green deep eutectic solvents and a new infrared technology. LWT.

[34] Castangia I., Manca M.L., Matricardi P., Sinico C., Lampis S., Fernàndez-Busquets X., Fadda A.M., Manconi M. (2013). Effect of diclofenac and glycol intercalation on structural assembly of phospholipid lamellar vesicles. Int. J. Pharm..

[35] Castangia I., Caddeo C., Manca M.L., Casu L., Latorre A.C., Díez-Sales O., Ruiz-Saurí A., Bacchetta G., Fadda A.M., Manconi M. (2015). Delivery of liquorice extract by liposomes and hyalurosomes to protect the skin against oxidative stress injuries. Carbohydr. Polym..

[36] Yue P.Y.K. (2010). A Simplified Method for Quantifying Cell Migration/Wound Healing in 96-Well Plates. J. Biomol. Screen..

[37] Manca M.L., Mir-Palomo S., Caddeo C., Nacher A., Díez-Sales O., Peris J.E., Pedraz J.L., Fadda A.M., Manconi M. (2019). Sorbitol-penetration enhancer containing vesicles loaded with baicalin for the protection and regeneration of skin injured by oxidative stress and UV radiation. Int. J. Pharm..

[38] Manca M.L., Castangia I., Zaru M., Nácher A., Valenti D., Fernàndez-Busquets X., Fadda A.M., Manconi M. (2015). Development of curcumin loaded sodium hyaluronate immobilized vesicles (hyalurosomes) and their potential on skin inflammation and wound restoring. Biomaterials.

[39] Akgün D., Gültekin M.-O., Yücetepe A., Altin G., Gibis M., Weiss J. (2020). Food Hydrocolloids Stirred-type yoghurt incorporated with sour cherry extract in chitosan-coated liposomes. Food Hydrocoll..

[40] Catalán-Latorre A., Pleguezuelos-Villa M., Castangia I., Manca M.L., Caddeo C., Nácher A., Díez-Sales O., Peris J.E., Pons R., Escribano-Ferrer E. (2018). Nutriosomes: Prebiotic delivery systems combining phospholipids, a soluble dextrin and curcumin to counteract intestinal oxidative stress and inflammation. Nanoscale.

[41] Manconi M., Mura S., Manca M.L., Fadda A.M., Dolz M., Hernandez M.J., Casanovas A., Diez-Sales O. (2010). Chitosomes as drug delivery systems for C-phycocyanin: Preparation and characterization. Int. J. Pharm..

[42] Atrooz O.M. (2007). The incorporation effects of methanolic extracts of some plant seeds on the stability of phosphatidylcholine liposomes. Pak. J. Biol. Sci..

[43] Fang Z. (2010). Encapsulation of polyphenols: A review. Trends Food Sci. Technol..

[44] Krämer S.D., Jakits-Deiser C., Wunderli-Allenspach H. (1997). Free fatty acids cause pH-dependent changes in drug-lipid membrane interactions around physiological pH. Pharm. Res..

[45] Manca M.L., Peris J.E., Melis V., Valenti D., Cardia M.C., Lattuada D., Escribano-Ferrer E., Fadda A.M., Manconi M. (2015). Nanoincorporation of curcumin in polymer-glycerosomes and evaluation of their in vitro-in vivo suitability as pulmonary delivery systems. RSC Adv..

[46] Murthy S.N., Zhao Y.L., Sen A., Hui S.W. (2004). Cyclodextrin enhanced transdermal delivery of piroxicam and carboxyfluorescein by electroporation. J. Control. Release.

[47] Lewis S., Pandey S., Udupa N. (2006). Design and evaluation of matrix type and membrane controlled transdermal delivery systems of nicotine suitable for use in smoking cessation. Indian J. Pharm. Sci..

[48] Allen T.M., Moase E.H. (1996). opportunities for targeted liposomal drug delivery. Adv. Drug Deliv. Rev..

[49] Chithrani D.B., Dunne M., Stewart J., Allen C., Jaffray D.A. (2010). Cellular uptake and transport of gold nanoparticles incorporated in a liposomal carrier. Nanomed. Nanotechnol. Biol. Med..

[50] Pes I., Baykal T., Alper M., Yes E. (2010). Investigations on the in vivo wound healing potential of *Hypericum perforatum* L.. Ipek Pes..

[51] Mukherjee P.K., Verpoorte R., Suresh B. (2000). Evaluation of in-vivo wound healing activity of Hypericum patulum (Family: Hypericaceae) leaf extract on different wound model in rats. J. Ethnopharmacol..

[52] Mukherjee P.K., Suresh B. (2000). The evaluation of wound-healing potential of Hypericum hookerianum leaf and stem extracts. J. Altern. Complement. Med..

[53] Süntar I.P., Akkol E.K., Yilmazer D., Baykal T., Kirmizibekmez H., Alper M., Yeşilada E. (2010). Investigations on the in vivo wound healing potential of *Hypericum perforatum* L.. J. Ethnopharmacol..

[54] Orčić D.Z., Mimica-duki N.M., Franci M.M., Petrovi S.S., Jovin E.Đ. (2011). Antioxidant activity relationship of phenolic compounds in *Hypericum perforatum* L.. Chem Cent J..

[55] Pistelli L., Bertoli A., Zucconelli S., Morelli I. (2000). Antimicrobial activity of crude extracts and pure compounds of *Hypericum hircinum*. Fitoterapia.

[56] Pillaiyar T., Manickam M., Namasivayam V. (2017). Skin whitening agents: Medicinal chemistry perspective of tyrosinase inhibitors. J. Enzym. Inhib. Med. Chem..

[57] Cai M.S., Xing J., Corke H. (2004). Antioxidant Phenolic Constituents in Roots of Rheum officinale and Rubia cordifolia: Structure—Radical Scavenging Activity Relationships. J. Agric. Food Chem..

[58] Wittenauer J., Mäckle S., Sußmann D., Schweiggert-weisz U., Carle R. (2015). Inhibitory effects of polyphenols from grape pomace extract on collagenase and elastase activity. Fitoterapia.

[59] Genc Y., Dereli F.T.G., Saracoglu I., Kupeli E. (2020). The inhibitory effects of isolated constituents from *Plantago major* subsp. major L. on collagenase, elastase and hyaluronidase enzymes: Potential wound healer. Saudi Pharm. J..

[60] Derycke A.S.L., De Witte P.A.M. (2002). Transferrin-mediated targeting of hypericin embedded in sterically stabilized PEG-liposomes. Int. J. Oncol..

[61] Plenagl N., Seitz B.S., Duse L., Pinnapireddy S.R., Jedelska J., Brüßler J., Bakowsky U. (2019). Hypericin inclusion complexes encapsulated in liposomes for antimicrobial photodynamic therapy. Int. J. Pharm..

[62] Manca M.L., Matricardi P., Cencetti C., Peris J.E., Melis V., Carbone C., Escribano E., Zaru M., Fadda A.M., Manconi M. (2016). Combination of argan oil and phospholipids for the development of an effective liposome-like formulation able to improve skin hydration and allantoin dermal delivery. Int. J. Pharm..

[63] Yang S., Liu L., Han J., Tang Y. (2020). Encapsulating plant ingredients for dermocosmetic application: An updated review of delivery systems and characterization techniques. Int. J. Cosmet. Sci..

[64] Saravanakumar K., Hu X., Chelliah R., Oh D. (2020). Biogenic silver nanoparticles-polyvinylpyrrolidone based glycerosomes coating to expand the shelf life of fresh-cut bell pepper (*Capsicum annuum* L.. var. grossum (L.) Sendt). Postharvest Biol. Technol..

[65] Mir-Palomo S., Nácher A., Díez-Sales O., Buso M.A.O., Caddeo C., Manca M.L., Manconi M., Fadda M.A. (2016). Inhibition of skin in fl ammation by baicalin ultradeformable vesicles. Int. J. Pharm..

[66] Moolakkadath T., Aqil M., Ahad A., Imam S.S., Praveen A., Sultana Y., Mujeeb M. (2020). Preparation and optimization of fi setin loaded glycerol based soft nanovesicles by Box-Behnken design. Int. J. Pharm..

[67] Manca M.L., Cencetti C., Matricardi P., Castangia I., Zaru M., Sales O.D., Nacher A., Valenti D., Maccioni A.M., Fadda A.M. (2016). Glycerosomes: Use of hydrogenated soy phosphatidylcholine mixture and its effect on vesicle features and diclofenac skin penetration. Int. J. Pharm..

[68] Manca M.L., Castangia I., Caddeo C., Pando D., Escribano E., Valenti D., Lampis S., Zaru M., Fadda A.M., Manconi M. (2014). Improvement of quercetin protective effect against oxidative stress skin damages by incorporation in nanovesicles. Colloids Surf. B Biointerfaces.

[69] Zhang K., Li N. (2017). Essential oil-mediated glycerosomes increase transdermal paeoniflorin delivery: Optimization, characterization, and evaluation in vitro and in vivo. Int. J. Nanomed..

[70] Manconi M., Manca M.L., Valenti D., Escribano E., Hillaireau H., Fadda A.M., Fattal E. (2017). Chitosan and hyaluronan coated liposomes for pulmonary administration of curcumin. Int. J. Pharm..

[71] Manconi M., Manca M.L., Marongiu F., Caddeo C., Castangia I., Petretto G.L., Pintore G., Sarais G., D’Hallewin G., Zaru M. (2016). Chemical characterization of *Citrus limon* var. pompia and incorporation in phospholipid vesicles for skin delivery. Int. J. Pharm..

[72] Dong C., Rogers J.A. (1993). Acacia-Gelatin Microencapsulated Liposomes: Preparation, Stability, and Release of Acetylsalicylic Acid. Pharm. Res..

[73] DiTizio V., Karlgard C., Lilge L., Khoury A.E., Mittelman M.W., DiCosmo F. (2000). Localized drug delivery using crosslinked gelatin gels containing liposomes: Factors influencing liposome stability and drug release. J. Biomed. Mater. Res..

[74] Shishova N.V., Davydova G.A., Kombarova N.A., Poltavtsev A.M., Zaraisky E.I., Poltavtseva R.A. (2020). The use of nanosized liposomes from vegetable phospholipids in combination with albumine and some polysaccharides as cryoprotective agents in the course of cryopreservation. IOP Conf. Ser. Mater. Sci. Eng..

[75] Manconi M., Caddeo C., Manca M.L., Fadda A.M. (2020). Oral delivery of natural compounds by phospholipid vesicles. Nanomedicine.

[76] Hu F., Yan Y., Wang C., Liu Y., Wang J., Zhou F., Zeng Q., Zhou X., Chen J., Wang A. (2019). Article Effect and Mechanism of *Ganoderma lucidum* Polysaccharides on Human Fibroblasts and Skin Wound Healing in Mice. Chin. J. Integr. Med..

[77] Kumar S., Marrero-Berrios I., Kabat M., Berthiaume F. (2019). Recent advances in the use of algal polysaccharides for skin wound healing. Curr. Pharm. Des..

[78] Kumar S., Dutta J., Dutta P.K., Koh J. (2020). A systematic study on chitosan-liposome based systems for biomedical applications. Int. J. Biol. Macromol..

[79] Manca M.L., Casula E., Marongiu F., Bacchetta G., Sarais G., Zaru M., Escribano-Ferrer E., Peris J.E., Usach I., Fais S. (2020). From waste to health: Sustainable exploitation of grape pomace seed extract to manufacture antioxidant, regenerative and prebiotic nanovesicles within circular economy. Sci. Rep..

[80] Manconi M., Marongiu F., Manca M.L., Caddeo C., Sarais G., Cencetti C., Pucci L., Longo V., Bacchetta G., Fadda A.M. (2017). Nanoincorporation of bioactive compounds from red grape pomaces: In vitro and ex vivo evaluation of antioxidant activity. Int. J. Pharm..

